# Dr. Westmoreland and Atlanta Medical College

**Published:** 1882-07-20

**Authors:** J. G. Westmoreland


*DR. JNO. G. WESTMNRELAND AND THE AILANTA MEDICAL COLLEGE.*

The following card appeared in the Atlanta Constitution of July 2d, 1882:

TO THE PUBLIC.

More than a year ago I resigned my chair of Materia Medica and Therapeutics in the Atlanta Medical College, which I had occupied from the organization in 1855. By that act, as the Faculty were well apprised, my connection with the Institution was nominally and practically severed. In the succeeding catalogue my name was retained as Emeritus Professor, as I supposed to show an honorable separation from the Faculty, and of course was not objected to. It is, however, inserted again in the catalogue of the present year, without my knowledge, desire or consent, or any valid reason, as I conceive.

My resignation was founded on objections to the policy adopted by the Faculty of taxing the students and Faculty with full fees for a young man to act as clerk for the Dean and whipper-in for the College, and I do not, therefore, intend to retain even a nominal connection, though nearly a quarter of a century of the prime of my life was spent in the organization and suecessful operation of the College. If the continuance of my name is intended as an honor to me, I respectfully decline it, and if for other purposes my duty to the public requires this notice.

J. G. WESTMORELAND.

